# The oncogenic role of tubulin alpha-1c chain in human tumours

**DOI:** 10.1186/s12885-022-09595-0

**Published:** 2022-05-06

**Authors:** Xinyao Hu, Hua Zhu, Biao Chen, Xiaoqin He, Yang Shen, Xiaoyu Zhang, Yangtao Xu, Ximing Xu

**Affiliations:** 1 Cancer Center, Renmin Hospital of Wuhan University, 99 Zhangzhidong Road, Wuchang District, Wuhan, 430060 Hubei Province China; 2 Department of Neurosurgery, Renmin Hospital of Wuhan University, 99 Zhangzhidong Road, Wuchang District, Wuhan, 430060 Hubei Province China

**Keywords:** TUBA1C, Pancancer, Tumour immunity, Prognosis, Immune infiltration

## Abstract

Tubulin alpha-1c chain (TUBA1C), a subtype of α-tubulin, has been shown to be involved in cell proliferation and cell cycle progression in several cancers and to influence cancer development and prognosis. However, a pancancer analysis of TUBA1C to reveal its immunological and prognostic roles has not been performed. In this study, we first downloaded raw data on TUBA1C expression in cancers from The Cancer Genome Atlas (TCGA) database and multiple other databases and analysed these data with R software to investigate the prognostic and immunological value of TUBA1C in cancers. Immunohistochemical analysis was performed in gliomas to further validate our findings. Overall, TUBA1C was overexpressed in most cancers, and overexpression of TUBA1C was linked to poor prognosis and higher tumour grade in patients. In addition, TUBA1C expression was associated with tumour mutation burden (TMB), microsatellite instability (MSI), the tumour microenvironment (TME) and the infiltration of immune cells. TUBA1C was also coexpressed with most immune-related genes and influenced immune-related pathways. Immunohistochemical analysis showed that TUBA1C expression was highest in glioblastoma (GBM) tissues, second highest in low-grade glioma (LGG) tissues and lowest in normal tissues. Our study indicated that TUBA1C might be a biomarker for predicting the immune status and prognosis of cancers, offering new ideas for cancer treatment.

**Supplementary Information:**

The online version contains supplementary material available at 10.1186/s12885-022-09595-0.

## Introduction

Cancer, as a leading cause of death worldwide, is currently a major obstacle to increasing life expectancy, with its morbidity and mortality rates rising rapidly [1]. The common therapeutic regimens for patients with advanced cancers, mainly including surgery, radiotherapy, chemotherapy and biologic therapies, are still fall short of expectations [2]. Thus, it is absolutely imperative to identify new effective treatments to improve patient prognosis and quality of life. Cancer immunotherapy, dedicated to reactivating the antitumor immune response and blocking pathways that lead to immune escape, has received increasing attention; immune checkpoint blockade inhibitors (ICIs) have especially been noticed [3].

Microtubules are essential cytoskeletal components that play a key role in cell division, generation, motility and intracellular transport. Polymeric microtubules are assembled from the highly conserved subunits α- and β-tubulin [4]. Tubulin alpha-1c chain (TUBA1C) is an isoform of α-tubulin. It has been demonstrated that TUBA1C plays a significant role in the cell cycle and immune microenvironment in lung adenocarcinoma (LUAD). Elevated expression of TUBA1C was correlated with poor outcome and with 13 tumour-infiltrating immune cells (TIICs) in LUAD [5]. TUBA1C was found to be upregulated in hepatocellular carcinoma (HCC) and pancreatic ductal adenocarcinoma (PDAC), where it predicted poor prognosis and facilitated cell proliferation and migration [6, 7]. In addition, a previous study claimed that TUBA1C was statistically linked to the expression of RP11-480I12.5 in breast cancer (BRCA) and had prognostic value [8]. TUBA1C has also been shown to promote aerobic glycolysis and cell growth through upregulation of YAP expression, thereby playing a role in the development of BRCA [9]. Moreover, TUBA1C has been reported to be associated with glioma [10, 11]. These studies indicate that the TUBA1C gene has potential as a biomarker for tumour prognosis and immunotherapy outcomes. However, there has been no pancancer analysis of TUBA1C to date.

There is a complex link between malignant cancers and the tumour microenvironment (TME), which includes tumour cells, immune cells, stromal cells, endothelial cells, and cancer-associated fibroblasts [12]. Immunotherapy focuses on recognizing and attacking cancer cells through immune cells within and outside the TME. ICIs, mainly including Programmed cell death protein 1 (PD-1)/PD-1 ligand (PD-L1) and Cytotoxic T lymphocyte antigen-4 (CTLA-4) inhibitors, act primarily by preventing the inhibition of interactions between T cells and other cells or tissues, thus allowing uninhibited activation of T cells, resulting in an antitumor effect [13, 14]. Ipilimumab, an anti-CTLA-4 antibody, was the first ICI cleared for therapeutic use by the Food and Drug Administration (FDA) in 2011 [15]. The PD-1 blocking antibody pembrolizumab was approved by the FDA in 2014 for the treatment of patients with refractory myeloma [16]. The use of chemotherapy in combination with ICI also holds good therapeutic promise. However, research into immunotherapy for various cancers is still in its initial stages, and further research of more general or specific immune targets is still needed to improve patient prognosis. Both tumour mutational burden (TMB) and the microsatellite instability (MSI) status can reflect the immune response to predict the outcome of cancers [17, 18].

In the current study, we performed a systematic pancancer analysis of TUBA1C through databases such as Oncomine, CCLE, HPA, and TGCA to analyse its effects on cancer prognosis, clinicopathology, the immune response, and the tumour microenvironment and validated the findings by immunohistochemistry. The potential of TUBA1C as a new immunotherapeutic target for tumour therapy was revealed.

## Methods

### Data download and differential analysis of TUBA1C expression in cancers

Thirty-three cancer-related RNA sequencing datasets and the clinicopathological and survival data for the corresponding patients were downloaded from the UCSC Xena website (https://xena.ucsc.edu/, derived from TCGA). Next, we analysed the mRNA expression of TUBA1C in 33 human malignancies using the online cancer microarray database Oncomine (https://www.oncomine.org/). We set the filter as we reported before [19]. Next, we used Perl software to extract and integrate the expression information of TUBA1C in 33 cancers in TCGA (https://tcga.xenahubs.net) using the “wilcox.test” function. The R package “ggpubr” was applied to draw a box plot. In addition, the Tumor Immune Estimation Resource (TIMER) database (https://cistrome.shinyapps.io/timer/) [20] and Gene Expression Profiling Interactive Analysis (GEPIA) (http://gepia.cancer-pku.cn/) database [21] were used for further profiling of TUBA1C expression in cancers. Moreover, we collected immunohistochemical data for cancer tissues using the HPA database (https://www.proteinatlas.org/) [22] and compared them with those for normal tissues to confirm the differential expression of TUBA1C in cancers at the protein level. In addition, the CGGA database (http://www.cgga.org.cn/) [23] was used to further investigate the expression levels and prognostic value of TUBA1C in gliomas.

### Correlation analysis of TUBA1C expression with clinicopathological features and survival in human cancers

The survival information obtained from the TCGA database corresponding to each sample was used to further analyse the relationships between TUBA1C expression and clinical outcomes, including overall survival (OS), disease-specific survival (DSS), disease-free interval (DFI), and progression-free interval (PFI). The results of survival analysis were visualized using forest plots and Kaplan–Meier (KM) curves.

### Relationship between TUBA1C expression and immunity

To analyse the association between TUBA1C and TMB and MSI, we first collated the information from the TCGA database using Perl software. Next, the command “cor.test”, based on Spearman’s method, was used, and the R package “fmsb” was utilized to plot radar plots. We then calculated the immune and stromal fractions using the ESTIMATE algorithm. Cell-type identification by estimating relative subsets of RNA transcripts (CIBERSORT) algorithm was applied to assess tumour purity and stromal/immune cell infiltration in tumour tissues (*n* = 33) according to expression file [24]. Subsequently, we evaluated the relationship between TUBA1C and TME or immune cell infiltration by applying the R packages “ggplot2”, “ggExtra” and ggpubr” (cut-off value *p* < 0.001). We further explored the associations between TUBA1C expression and immune cell infiltration with CIBERSORT, EPIC, TIMER, quanTIseq, xCELL and MCPcounter.

Next, CD274, CTLA4, HAVCR2, PDCD1, LAG3, SIGLEC15, PDCD1LG2, and TIGIT were chosen as immune checkpoint-related genes, and the expression data of these 8 transcripts were extracted. We used the R packages “ggplot2”, “immuneeconv” and “pheatmap” to assess immune checkpoint expression and the coexpression of TUBA1C with these immune checkpoints. The response to ICI therapy was predicted using the TIDE algorithm [25].

We downloaded the gene lists for immune activation, immunosuppression, chemokine receptor proteins, chemokines and Major histocompatibility complex (MHC) molecules from GSEA and processed the data in R. The results are shown in heatmaps.

### Biological significance of TUBA1C in tumours

We obtained the Gene Ontology (GO) and Kyoto Encyclopedia of Genes and Genomes (KEGG) gene sets [26] from GSEA (http://www.gsea-msigdb.org/gsea/downloads.jsp) to investigate the functional pathways of TUBA1C in tumours and then used the R packages “limma,” “org.Hs.eg.db,” “enrichplot,” and “clusterProfiler” to perform of GO functional and KEGG pathway annotation as well as pathway enrichment analysis of TUBA1C.

### Immunohistochemical analysis of glioma

We collected specimens for immunohistochemistry from low-grade glioma (LGG), glioblastoma (GBM) and normal tissues from Renmin Hospital of Wuhan University. After deparaffinization, hydration and antigen repair, endogenous peroxidase activity in the sections was inhibited with 3% hydrogen peroxide for 15 min and the sections were then blocked with BSA, incubated with an anti-TUBA1C primary antibody (ab222849) overnight at 4 °C, and incubated with a secondary antibody for 50 min. Colour was developed with DAB chromogen, and the sections were restained with haematoxylin and finally subjected to dehydration and sealing. Finally, images were acquired by microscopy. We used ImageJ to quantify the immunohistochemical results.

### Statistical analysis

The mRNA expression data of all genes in cancers were normalized using log2 transformation. Differences in data for each group were obtained using t tests or ANOVA. Significance was analysed with 0.05 as the threshold. Survival analysis was performed using Cox regression models, the KM method and the log-rank test. Moreover, Spearman’s or Pearson’s tests were used to identify relationships between two genes. All statistical procedures were completed in R software.

## Results

### TUBA1C expression levels across cancers

**Fig. 1:**
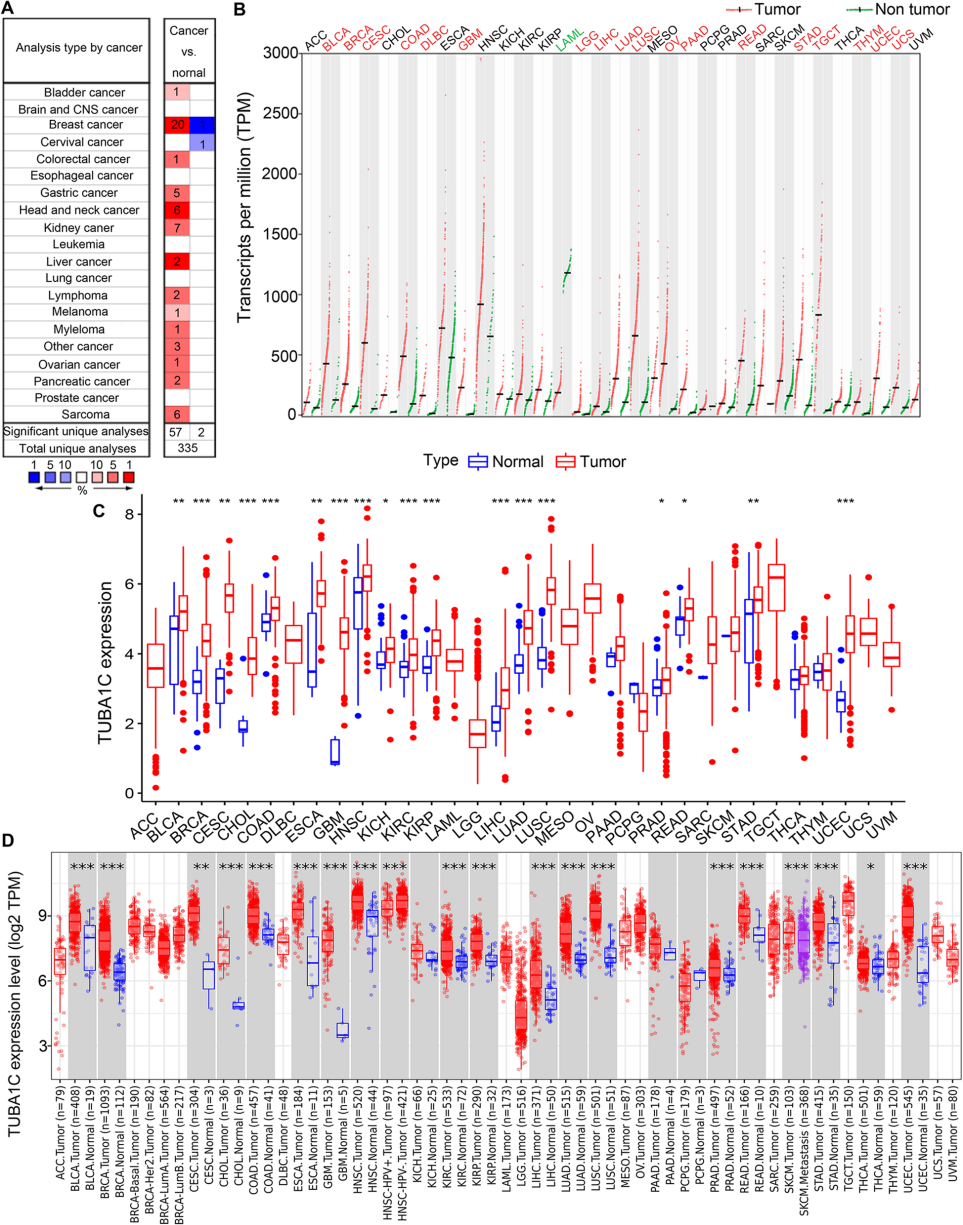
Differential TUBA1C expression in 33 cancers. **A** Differential expression of TUBA1C in cancerous and normal tissues analysed by Oncomine. **B** Differential expression of TUBA1C in cancers from GEPIA. **C** Analysis of the differential expression of TUBA1C in cancers from TCGA data. **D** Differential expression of TUBA1C in cancers in TIMER. *p < 0.05, **p < 0.01, ***p < 0.001

We used Oncomine to assess TUBA1C gene expression in 33 cancers. It was shown that TUBA1C expression was elevated in most tumours, including bladder cancer (BLCA), breast cancer (BRCA), colorectal cancer (COAD), gastric cancer, head and neck cancer (HNSC), kidney cancer, liver cancer (LIHC), lymphoma, melanoma, myeloma, ovarian cancer (OV), pancreatic cancer (PAAD), and sarcoma (SARC). In contrast, lower TUBA1C expression was also found in BRCA and cervical cancer (CESC) (Fig. 1A). We further explored the differential expression of TUBA1C in cancers in GEPIA. Similar to the results in Oncomine, TUBA1C was upregulated in most cancers, including BLCA, BRCA, CESC, COAD, large B-cell lymphoma (DLBC), GBM, LGG, LIHC, LUAD, lung squamous cell carcinoma (LUSC), OV, PAAD, rectal cancer (READ), stomach cancer (STAD), testicular cancer (TGCT), thymoma (THYM), endometrioid cancer (UCEC) and uterine carcinosarcoma (UCS). Intriguingly, lower expression of TUBA1C was noted in acute myeloid leukaemia (LAML) (Fig. 1B).

We then analysed the data from TCGA with R software. A total of 11,057 mRNA expression profiles were obtained for 33 cancer types comprising 730 normal samples and 10,327 tumour samples. We found that TUBA1C showed higher expression in tumour samples in BLCA, BRCA, CESC, bile duct cancer (CHOL), COAD, oesophageal cancer (ESCA), GBM, HNSC, kidney chromophobe (KICH), kidney clear cell carcinoma (KIRC), kidney papillary cell carcinoma (KIRP), LIHC, LUAD, LUSC, prostate cancer (PRAD), READ, STAD and UCEC. No low expression of TUBA1C was detected in 33 cancers (Fig. 1C). Subsequently, TUBA1C was found to be upregulated in BLCA, BRCA, CESC, CHOL, COAD, ESCA, GBM, KIRC, KIRP, LIHC, LUAD, LUSC, PRAD, READ, STAD, THCA and UCEC by analysis with TIMER. In addition, the TUBA1C expression level was higher in HNSC-HPV+ tumours than in HNSC-HPV- tumours, and it was higher in SKCM tumours than in SKCM metastatic tumours (Fig. 1D). In conclusion, TUBA1C functions as a tumour promoter in most cancer types.

**Fig. 2:**
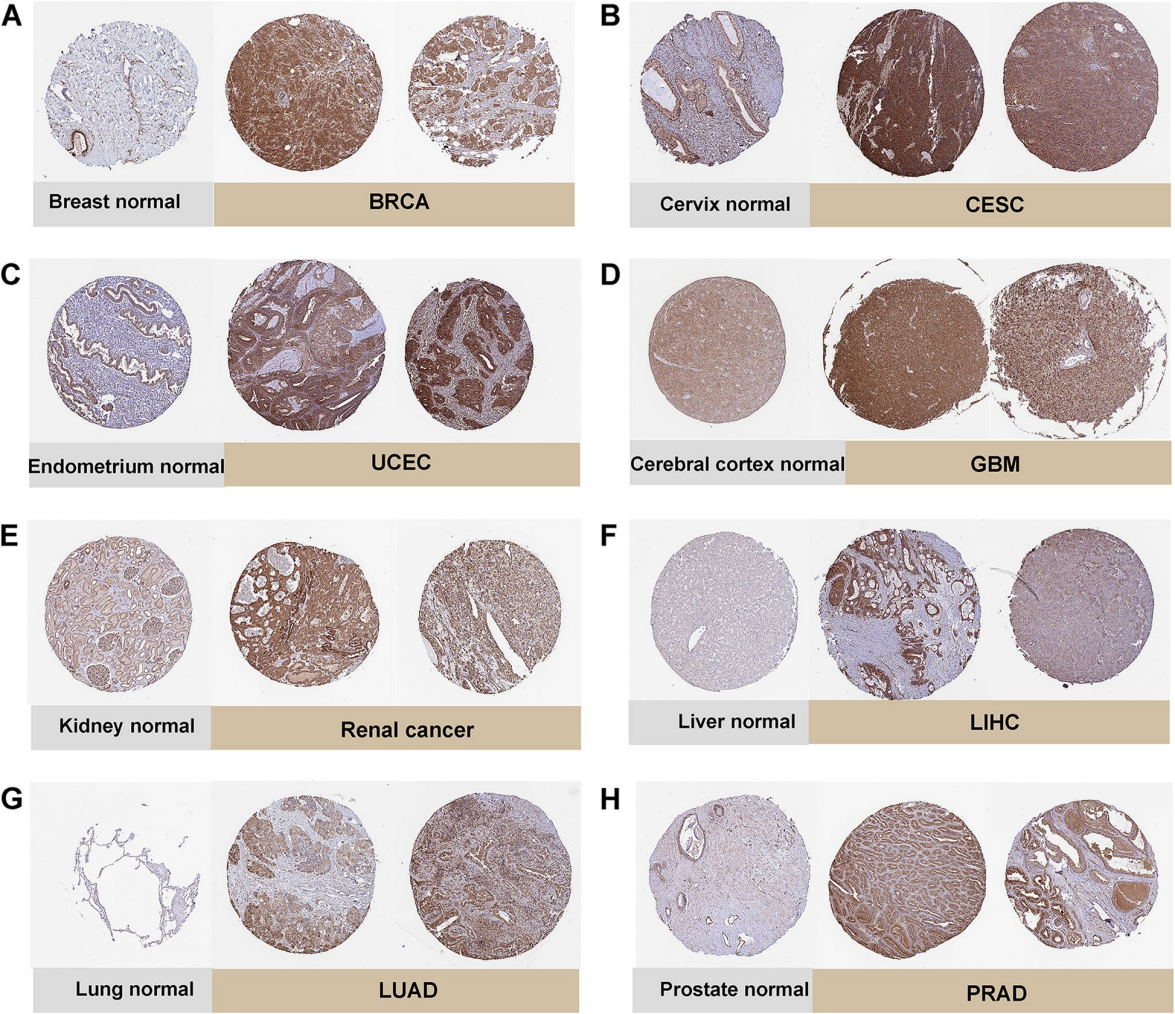
Immunohistochemical staining in normal tissues and tumour tissues from the HPA database. **A** Breast normal, BRCA. **B** Cervix normal, CESC. **C** Endometrium normal, UCEC. **D** Cerebral cortex normal, GBM. **E** Kidney normal, Renal cancer. **F** Liver normal, LIHC. **G** Lung normal, LUAD. **H** Prostate normal, PRAD

Immediately thereafter, we investigated the differential expression of TUBA1C at the protein level in cancerous and normal tissues in depth using Human Protein Atlas (HPA, https://www.proteinatlas.org/). The IHC results of BRCA, UCEC, renal cancer, LUAD, CESC, GBM, LIHC, PRAD and normal tissues were showed in Fig. 2A-H and these results were consisted with the expression data of TUBA1C mRNA in TCGA.

### Prognostic value of TUBA1C across cancers

**Fig. 3:**
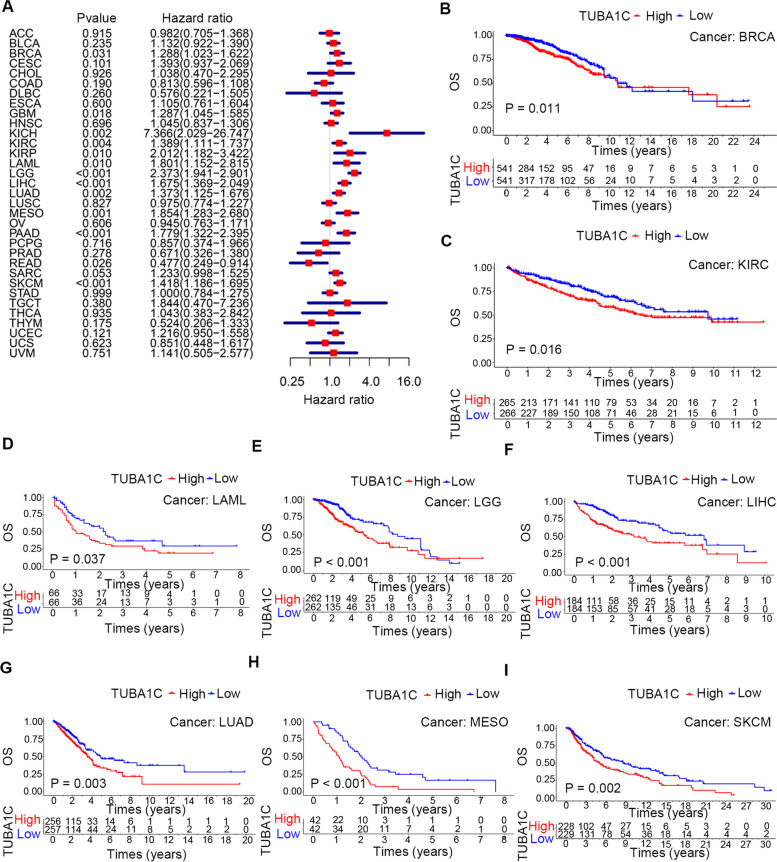
Association between TUBA1C expression and overall survival (OS) in patients. **A** Forest plot showing the HRs associated with TUBA1C expression in 33 cancer types. **B**-**I** KM OS curves for patients with different TUBA1C expression levels in BRCA, KIRC, LAML, LGG, LIHC, LUAD, MESO and SKCM

Subsequently, we explored the association between TUBA1C expression and the prognosis of patients across cancers. Cox regression analysis showed a significant relationship between TUBA1C expression and OS in BRAC (*P* = 0.031, HR = 1.288), GBM (*P* = 0.018, HR = 1.287), KICH (*P* = 0.002, HR = 7.366), KIRC (*P* = 0.004, HR = 1.389), KIRP (*P* = 0.01, HR = 2.012), LAML (*P* = 0.01, HR = 1.801), LGG (*P* < 0.001, HR = 2.373), LIHC (*P* < 0.001, HR = 1.675), LUAD (*P* = 0.002, HR = 1.373), MESO (*P* = 0.001, HR = 1.854), PAAD (*P* < 0.001, HR = 1.779), READ (*P* = 0.026, HR = 0.477) and SKCM (*P* < 0.001, HR = 1.418) (Fig. 3A). Moreover, our results suggested that high TUBA1C expression was a high-risk indicator for KIRC, KIRP, LAML, LGG, LIHC, LUAD, MESO, PAAD, SARC, and SKCM—especially KICH (HR = 7.366)—but was not a low-risk indicator for any cancer. In addition, a negative relationship between TUBA1C expression and OS was observed in 8 cancers (Fig. 3B-I): BRCA (*p* = 0.011), KIRC (*p* = 0.016), LAML (*p* = 0.037), LGG (*p* < 0.001), LIHC (*p* < 0.001), LUAD (*p* = 0.003) and SKCM (*p* = 0.002).

**Fig. 4:**
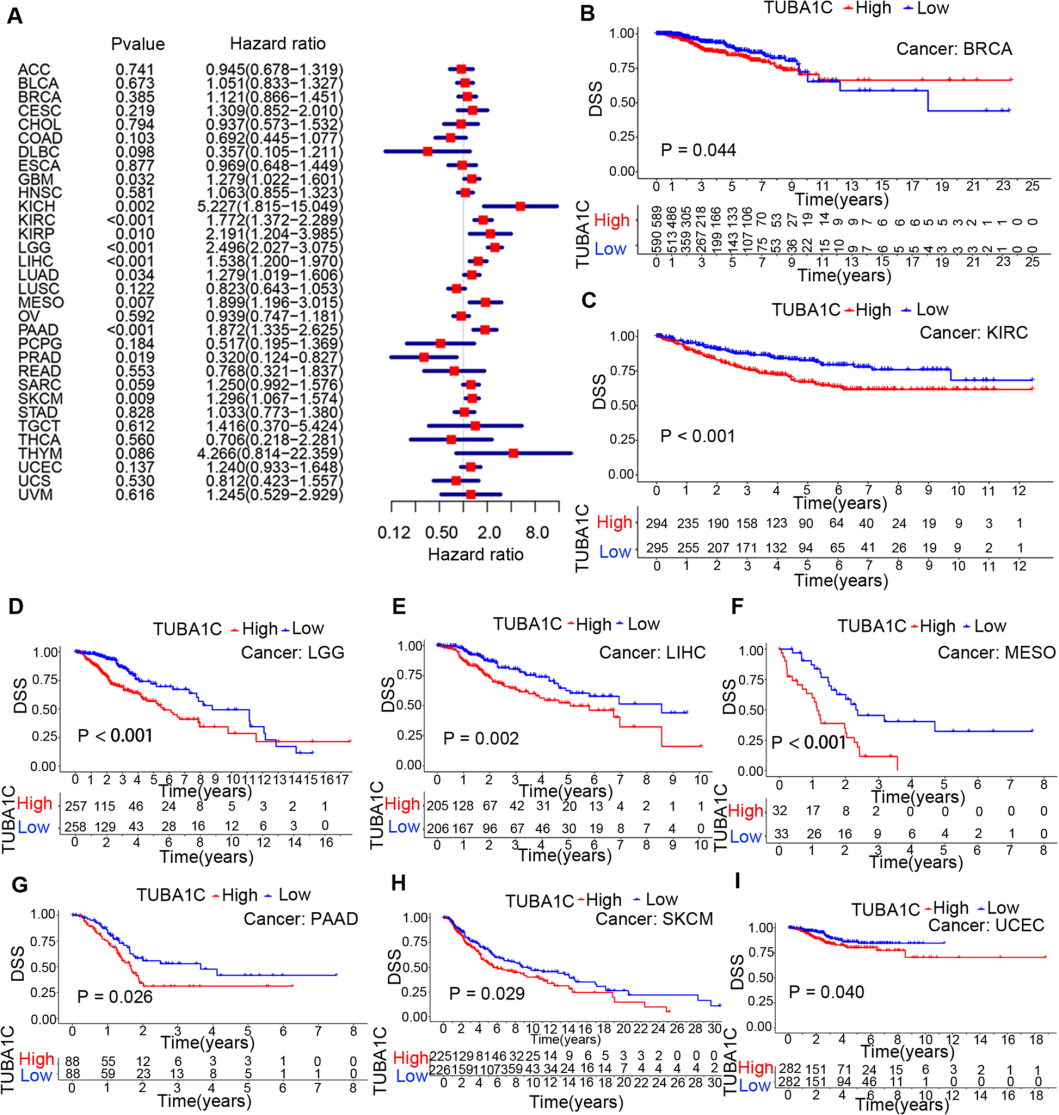
Association between TUBA1C expression and disease-specific survival (DSS) in patients. **A** Forest plot showing the HRs associated with TUBA1C expression in 33 cancer types. **B-I** KM DSS curves for patients with different TUBA1C expression levels in BRCA, KIRC, LGG, LIHC, MESO, PAAD, SKCM and UCEC

Since factors unrelated to the tumours may also contribute to death during follow-up, the relationship between TUBA1C expression and DSS in patients was subsequently analysed. It was revealed that TUBA1C expression impacted DSS in GBM (*p* = 0.032, HR = 1.279), KICH (*p* = 0.002, HR = 5.227), KIRC (*p* < 0.001, HR = 1.772), KIRP (*p* = 0.010, HR = 2.191), LGG (*p* < 0.001, HR = 2.498), LIHC (*p* < 0.001, HR = 1.538), LUAD (*p* = 0.034, HR =1.279), MESO (*p* = 0.007, HR = 1.899), PAAD (*p* < 0.001, HR =1.872), PRAD (*p* = 0.019, HR = 0.320) and SKCM (*p* = 0.009, HR = 1.296) (Fig. 4A). In these analyses, KICH displayed the highest HR (HR = 5.227). In addition, KM curves showed that high expression of TUBA1C was correlated with poor DSS in patients with BRCA (*p* = 0.044), KIRC (*p* < 0.001), LGG (*p* < 0.001), LIHC (*p* = 0.002), MESO (*p* < 0.001), PAAD (*p* = 0.026), SKCM (*p* = 0.029) and UCEC (*p* = 0.040) (Fig. 4B-I).

**Fig. 5:**
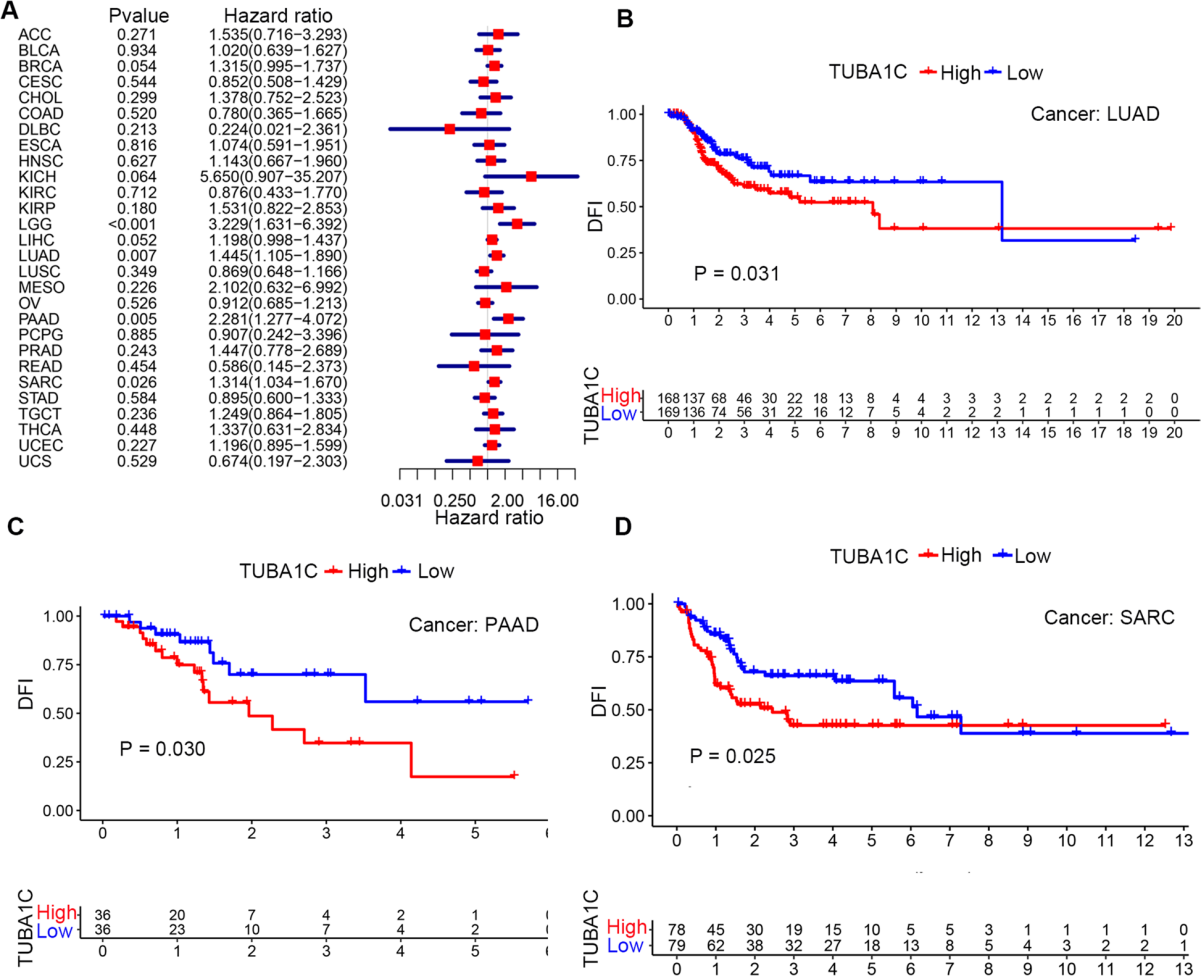
Association between TUBA1C expression and disease-free interval (DFI) in patients. **A** Forest plot showing the HRs associated with TUBA1C expression in 33 cancer types. **B-D** KM DFI curves for patients with different TUBA1C expression levels in LUAD, PAAD and SARC

**Fig. 6:**
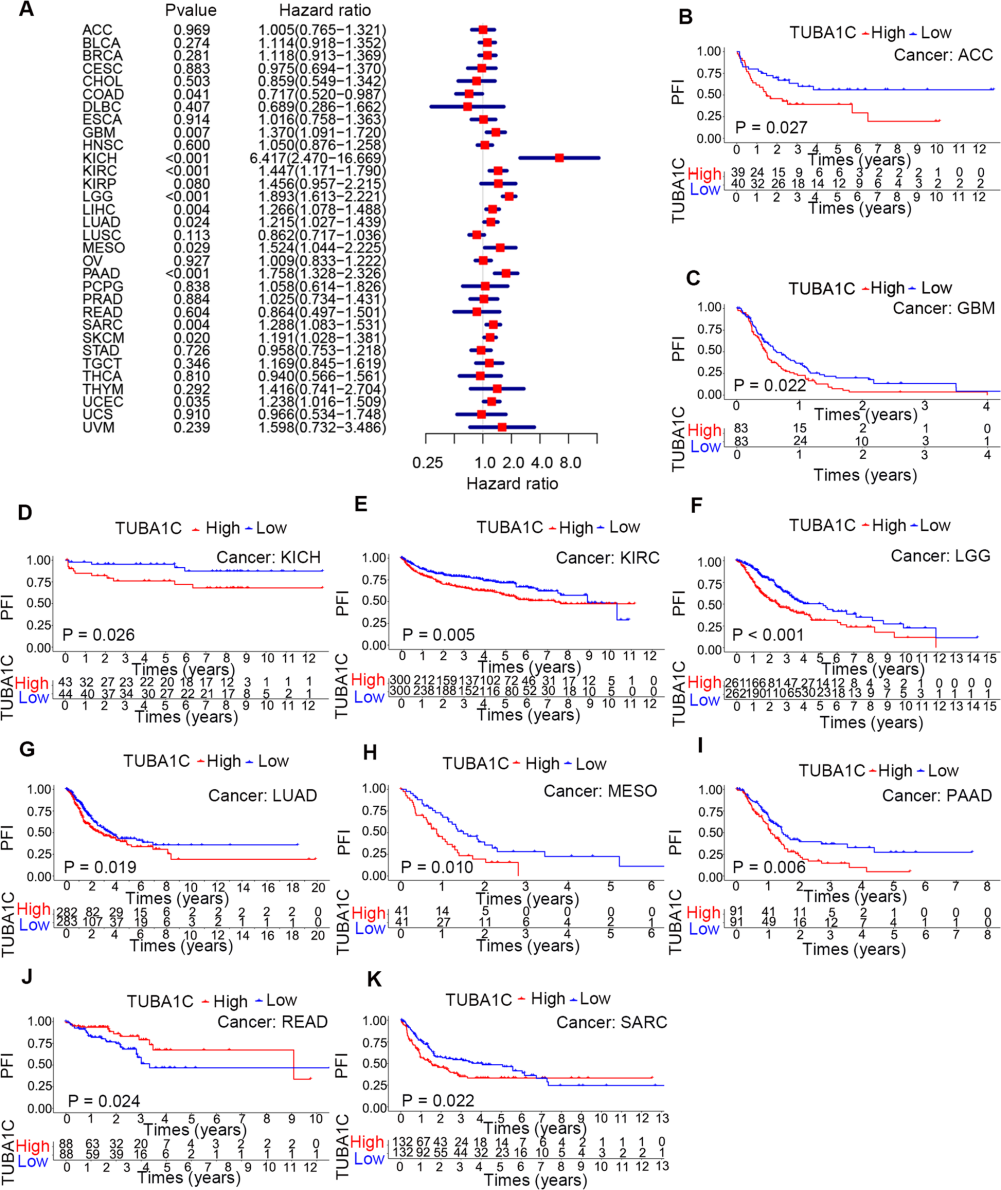
Association between TUBA1C expression and the progression-free interval (PFI) in patients. **A** Forest plot showing the HRs associated with TUBA1C expression in 33 cancer types. **B-K** KM PFI curves for patients with different TUBA1C expression levels in ACC, GBM, KICH, KIRC, LGG, LUAD, MESO, PAAD, READ and SARC

In addition, we explored the association between TUBA1C expression and the DFI in patients with different cancer types and found that increased TUBA1C expression was correlated with a decreased DFI in LGG (*p* < 0.001, HR = 3.229), LUAD (*p* = 0.007, HR = 1.445), PAAD (*p* = 0.005, HR = 2.281) and SARC (*p* = 0.026, HR = 1.314) (Fig. 5A). Moreover, in LUAD (*p* = 0.031), PAAD (*p* = 0.030) and SARC (*p* = 0.025), a high level of TUBA1C predicted a shorter DFI and poor prognosis in patients (Fig. 5B-D). Subsequently, we evaluated the association between TUBA1C expression and PFI. The forest plots showed that a high level of TUBA1C was correlated with a shorter PFI in COAD (*p* = 0.041, HR = 0.780), GBM (*p* = 0.007, HR = 1.893), KICH (*p* < 0.001, HR = 6.417), KIRC (*p* < 0.001, HR = 1.447), LGG (*p* < 0.001, HR = 1.893), LIHC (*p* = 0.004, HR = 1.266), LUAD (*p* = 0.024, HR = 1.215), MESO (*p* = 0.029, HR = 1.524), PAAD (*p* < 0.001, HR = 1.758), SARC (*p* = 0.004, HR = 1.288) and UCEC (*p* = 0.035, HR = 1.238). Among these cancers, KICH showed the highest HR (6.417, Fig. 6A). The results also indicated that high expression of TUBA1C affected the PFI unfavourably in ACC (*p* = 0.027) (Fig. 6B), GBM (*p* = 0.022) (Fig. 6C), KICH (*p* = 0.026) (Fig. 6D), KIRC (*p* = 0.005) (Fig. 6E), LGG (*p* < 0.001) (Fig. 6F), LUAD (*p* = 0.019) (Fig. 6G), MESO (*p* = 0.010) (Fig. 6H), PAAD (*p* = 0.006) (Fig. 6I) and SARC (*p* = 0.022) (Fig. 6K) but favourably in READ (*p* = 0.024) (Fig. 6J).

### Relationship between TUBA1C expression and clinicopathological stage in cancers

**Fig. 7:**
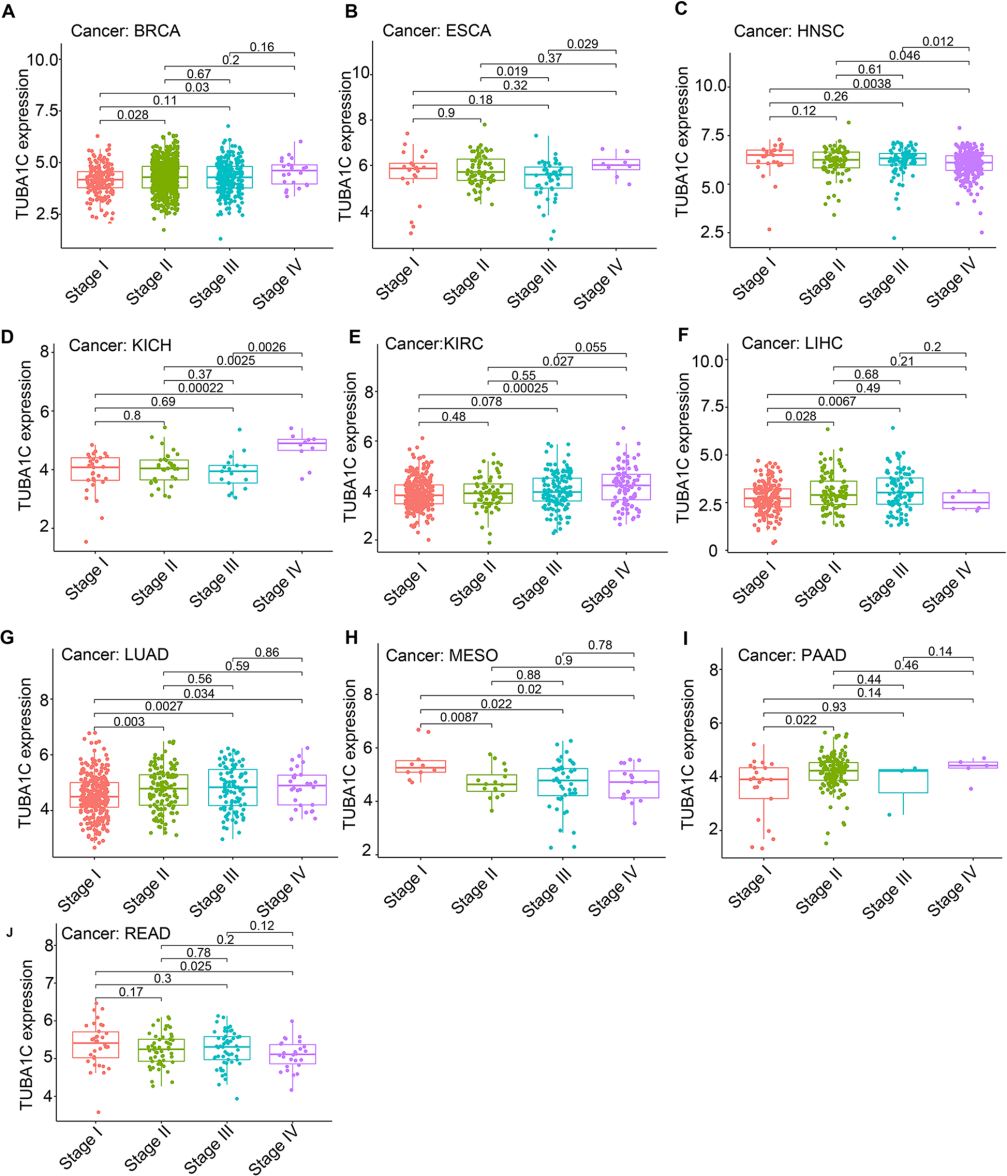
Relationship of TUBA1C expression with tumour stage in (**A**) BRCA, (**B**) ESCA, (**C**) HNSC, (**D**) KICH, (**E**) KIHC, (**F**) LIHC, (**G**) LUAD, (**H**) MESO, (**I**) PAAD and (**J**) READ

We next assessed the relationship between TUBA1C expression and tumour stage. We observed that in BRCA (Fig. 7A, *p* = 0.033), HNSC (Fig. 7C, *p* = 0.0038), KICH (Fig. 7D, *p* = 0.00022), KIRC (Fig. 7E, *p* = 0.00025), LUAD (Fig. 7G, *p* = 0.034), MESO (Fig. 7H, *p* = 0.02) and READ (Fig. 7J, *p* = 0.025), significant differences in TUBA1C expression existed between stage I and stage IV tumours. In LIHC (Fig. 7F, *p* = 0.0067), LUAD (Fig. 7G, *p* = 0.0027) and MESO (Fig. 7H, *p* = 0.022), the expression of TUBA1C was higher in stage III than in stage I tumours. In ESCA, the TUBA1C expression in stage IV was higher than in stage III (Fig. 7B, *p* = 0.029). Intriguingly, in PAAD (Fig. 7I, *p* = 0.022), TUBA1C expression was higher in stage II tumours than in stage I tumours, but no dramatic difference existed in stage III or IV tumours. In conclusion, TUBA1C expression was correlated with tumour stage.

### Relationship between TUBA1C expression and immunity

**Fig. 8:**
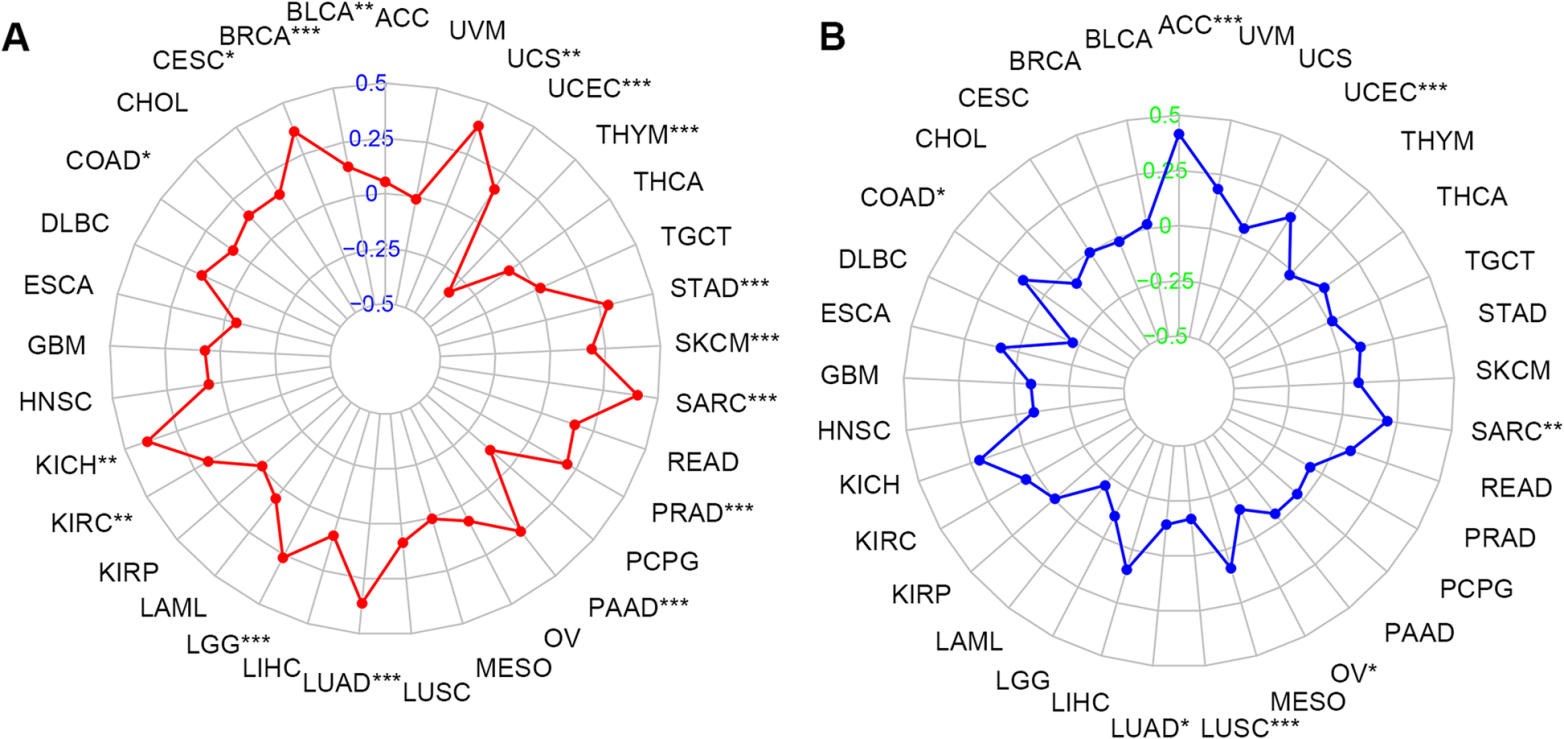
Relationships of TUBA1C expression with TMB and MSI in 33 cancer types. **A** Radar plot of the association of TUBA1C expression and TMB in 33 cancers. **B** Radar plot of the association of TUBA1C expression and MSI in 33 cancers

We first examined the association between TUBA1C expression level and TMB and MSI, two indicators that predict the sensitivity of cancer patients to ICIs. A positive association between TUBA1C expression and TMB was observed in 15 cancers, namely, UCS, UCEC, STAD, SKCM, SARC, PRAD, PAAD, LUAD, LGG, KIRC, KICH, COAD, CESC, BRCA and BLCA. In contrast, in THYM, a negative correlation between TUBA1C expression and TMB was observed (Fig. 8A). In ACC, UCEC, SARC and COAD, the TUBA1C expression level was positively associated with MSI, while in OV, LUSC, LUAD and LGG, a negative association existed between these factors (Fig. 8B).

**Fig. 9:**
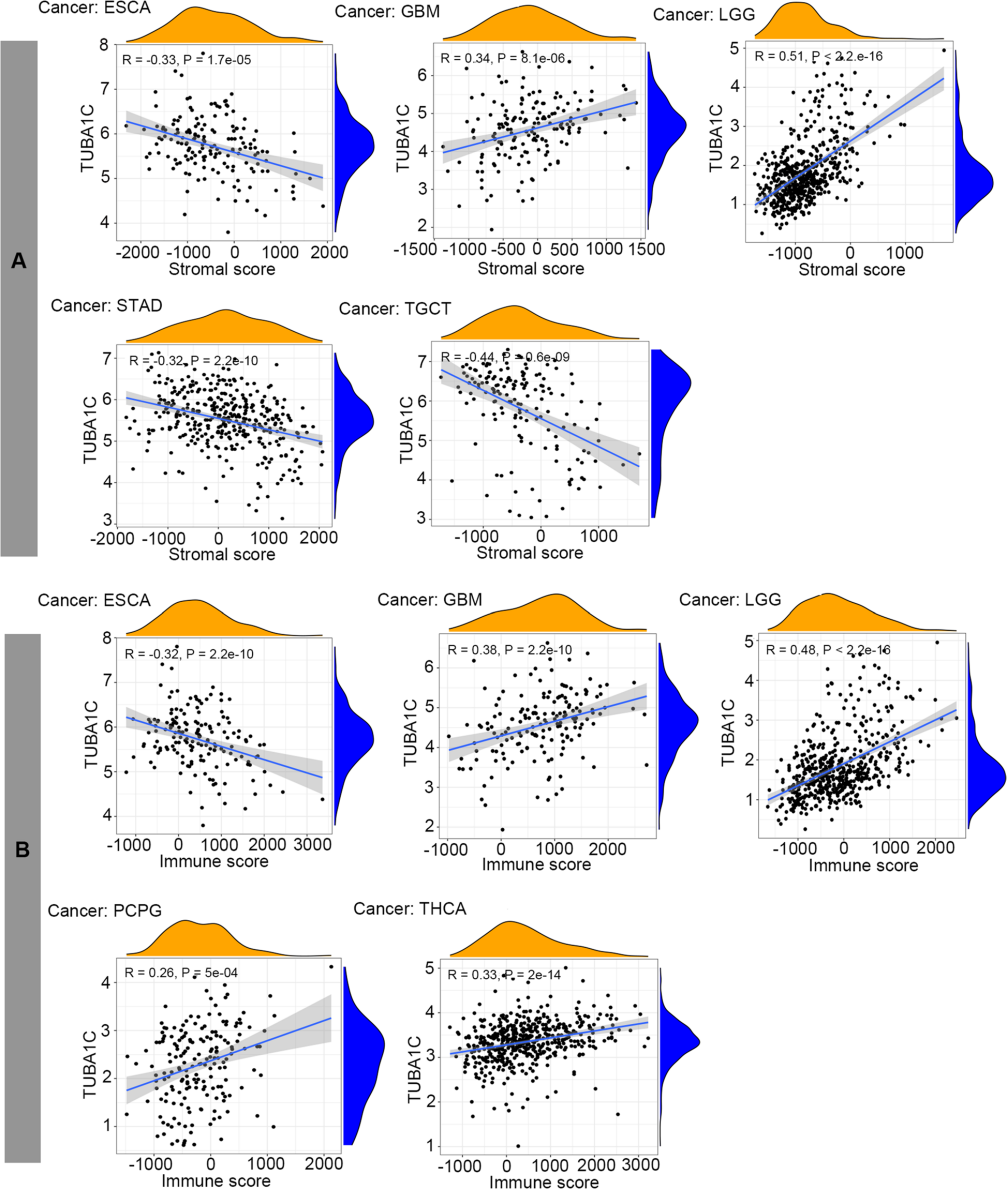
Relationships of TUBA1C expression with the TME. **A** TUBA1C expression was positively related to stromal scores in GBM and LGG and negatively related to the stromal score in ESCA, STAD and TGCT. **B** TUBA1C expression was positively related to immune scores in GBM, LGG, PCPG and THCA and negatively related to the immune score in ESCA

Tumour development is associated not only with abnormal mutations in tumour cells but also with the composition of their microenvironment and stromal cell proportions or activation states [27]. Therefore, the association between TUBA1C expression and the TME was investigated by assessing the relationships of stromal and immune scores to TUBA1C expression in 33 cancers using the ESTIMATE algorithm. It was shown that TUBA1C expression was positively correlated with stromal scores in GBM and LGG, while a negative correlation was observed in ESCA, STAD and TGCT (Fig. 9A). In addition, TUBA1C expression was positively related to immune scores in GBM, LGG, PCPG and THCA and negatively related to immune scores in ESCA (Fig. 9B).

**Fig. 10:**
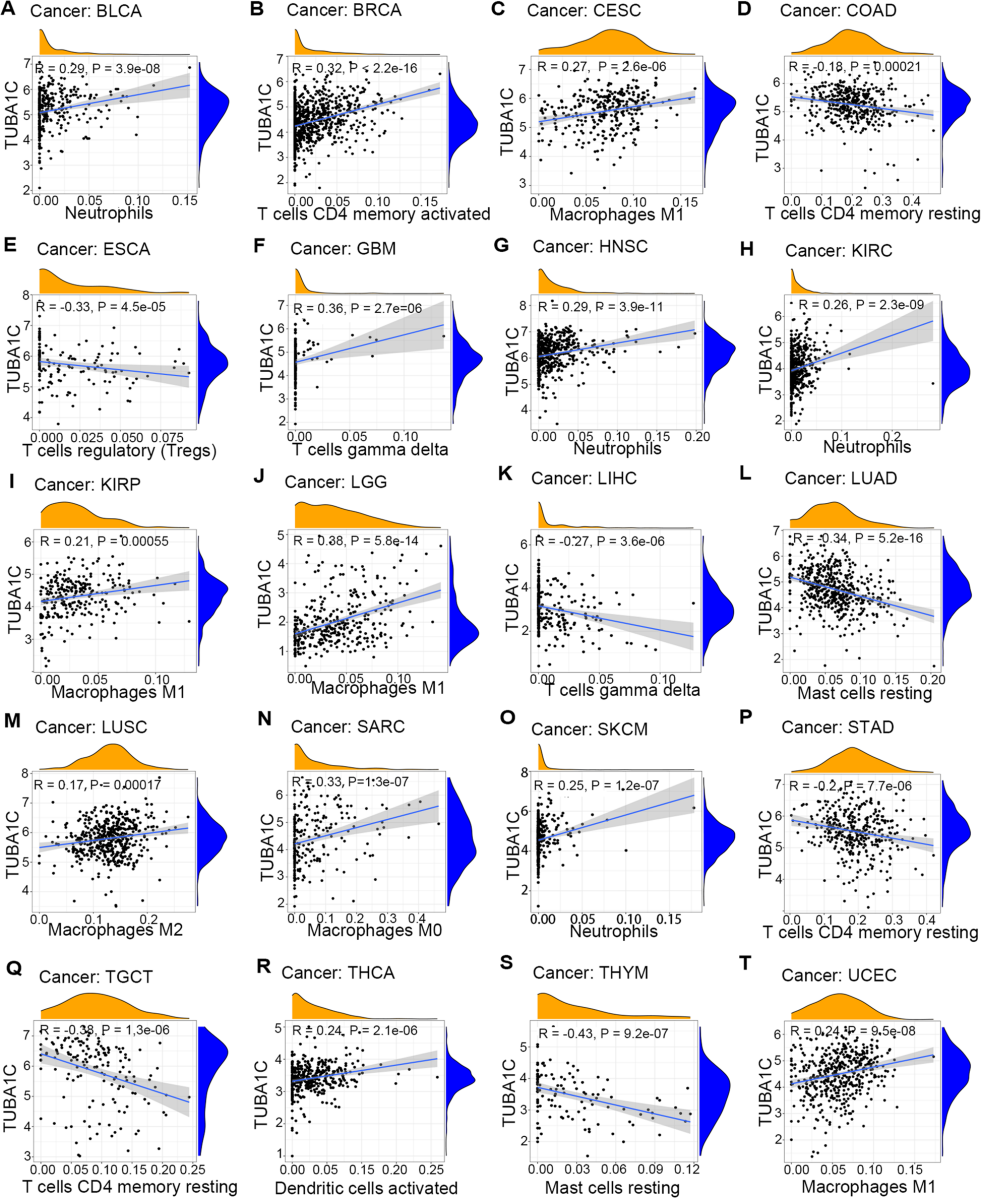
Relationships of TUBA1C expression with the infiltration of different immune cells in (**A**) BLCA, (**B**) BRCA, (**C**) CESC, (**D**) COAD, (**E**) ESCA, (**F**) GBM, (**G**) HNSC, (**H**) KIRC, (**I**) KIRP, (**J**) LGG, (**K**) LIHC, (**L**) LUAD, (**M**) LUSC, (**N**) SARC, (**O**) SKCM, (**P**) STAD, (**Q**) TGCT, (**R**) THCA, (**S**) THYM, and (**T**) UCEC

TIICs, an important component of the TME, are closely associated with the development, progression and metastasis of cancer. Herein, we examined the underlying relationship between the level of infiltration of 22 immune cell types and TUBA1C expression in different cancer types. We selected the TIICs with the highest correlation with TUBA1C expression in each cancer for demonstration (Fig. 10). Our results showed that TUBA1C expression was negatively related to the infiltration of memory CD4+ T cells in COAD (Fig. 10D), STAD (Fig. 10P) and TGCT (Fig. 10Q) but positively associated in BRCA (Fig. 10B). In ESCA (Fig. 10E), TUBA1C expression was observed to be negatively correlated with infiltration of regulatory T cells. In addition, TUBA1C expression was positively related to infiltration of neutrophils in BLCA (Fig. 10A), HNSC (Fig. 10G), KIRC (Fig. 10H) and SKCM (Fig. 10O) and positively correlated with infiltration of dendritic cells in THCA (Fig. 10R). In addition, the TUBA1C expression level was correlated with infiltration of several diverse subsets of macrophages in the TMB. TUBA1C expression was positively associated with M1 macrophage infiltration in CESC (Fig. 10C), KIRP (Fig. 10I), LGG (Fig. 10J) and UCEC (Fig. 10T), with M2 macrophage infiltration in LUSC (Fig. 10M) and with M0 macrophage infiltration in SARC (Fig. 10N). In addition, TUBA1C expression was negatively related to infiltration of mast cells in LUAD (Fig. 10L) and THYM (Fig. 10S). Moreover, TUBA1C expression was positively related to infiltration of gamma delta T cells in GBM (Fig. 10F) and LIHC (Fig. 10K). We performed further analyses with other algorithms, including CIBERSORT (Fig. S1), EPIC (Fig. S2), MCPcounter (Fig. S3), quanTIseq (Fig. S4), TIMER (Fig. S5) and xCELL (Fig. S6), and also found correlations between TUBA1C and immune cell infiltration.

**Fig. 11:**
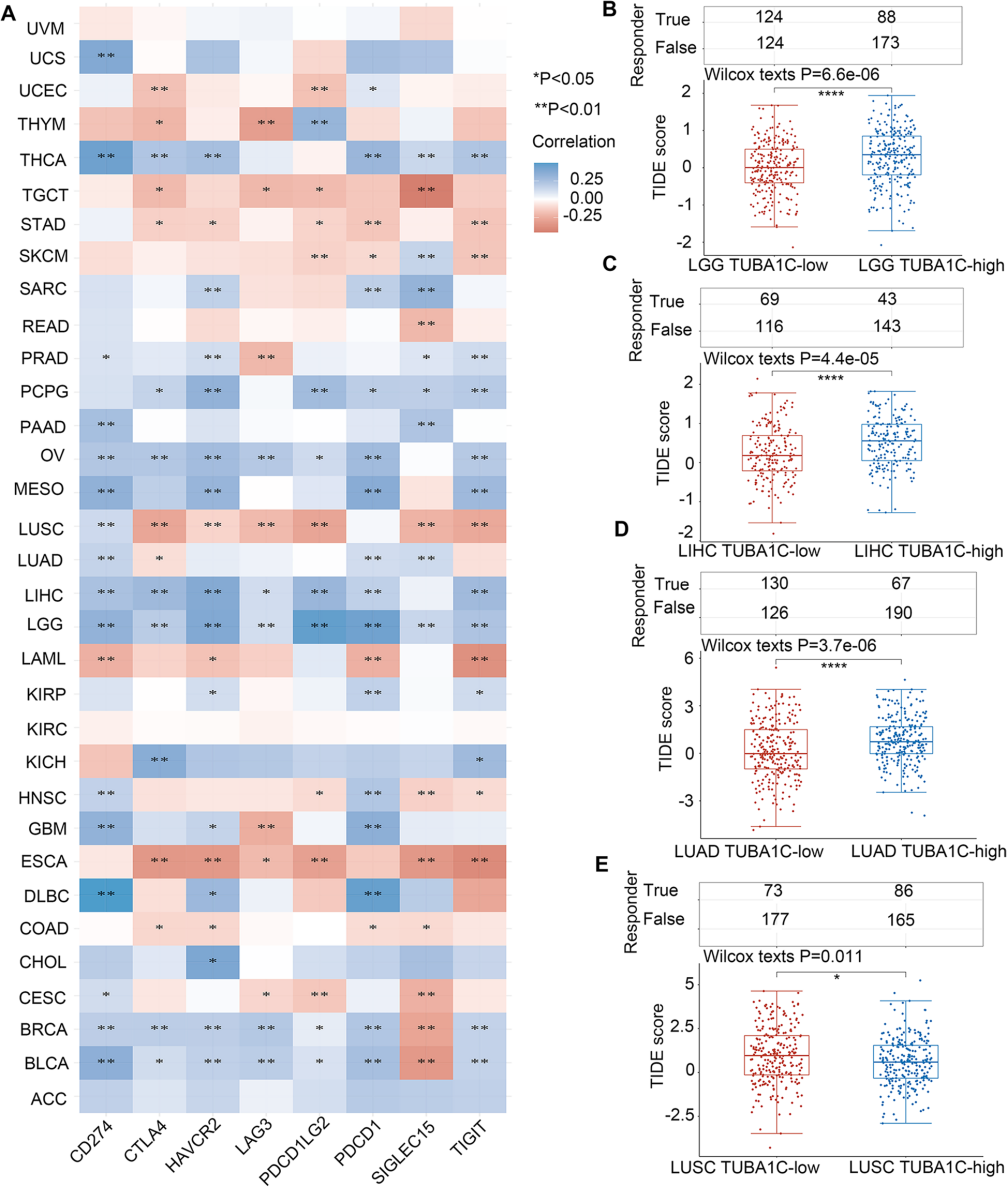
The relationship between TUBA1C and immune checkpoint expression and the patient response to ICI therapy. **A** TUBA1C was associated with immune checkpoints in most cancers. The TUBA1C-high group had a better response to ICI than the TUBA1C-low group in LGG (**B**), LIHC (**C**), LUAD (**D**), and LUSC (**E**)

Next, we investigated the correlations between TUBA1C expression and the expression of immune checkpoints, including CD274, HAVCR2, CTLA4, LAG3, PDCD1LG2, PDCD1, SIGLEC15, and TIGIT, in cancers. We found that TUBA1C was correlated with immune checkpoints in all cancers except UVM, KIRC and ACC. In LUSC and ESCA, TUBA1C expression was negatively related to the expression of most immune checkpoints, while in OV, LGG, BRCA and BLCA, a positive association of TUBA1C expression with immune checkpoints was revealed (Fig. 11A), suggesting that TUBA1C has promise as a predictive factor for immunotherapeutic response in patients with these cancers. Additionally, the group with high TUBA1C responded better to ICI therapy than the group with low TUBA1C in LGG (Fig. 11B, *p* < 0.001), LIHC (Fig. 11C, *p* < 0.001), LUAD (Fig. 11D, *p* < 0.001) and LUSC (Fig. 11E, *p* = 0.011), suggesting that TUBA1C has the potential to be an immunotherapy target.

### Relationship between TUBA1C expression and immune-associated genes and pathways in diverse cancers

**Fig. 12:**
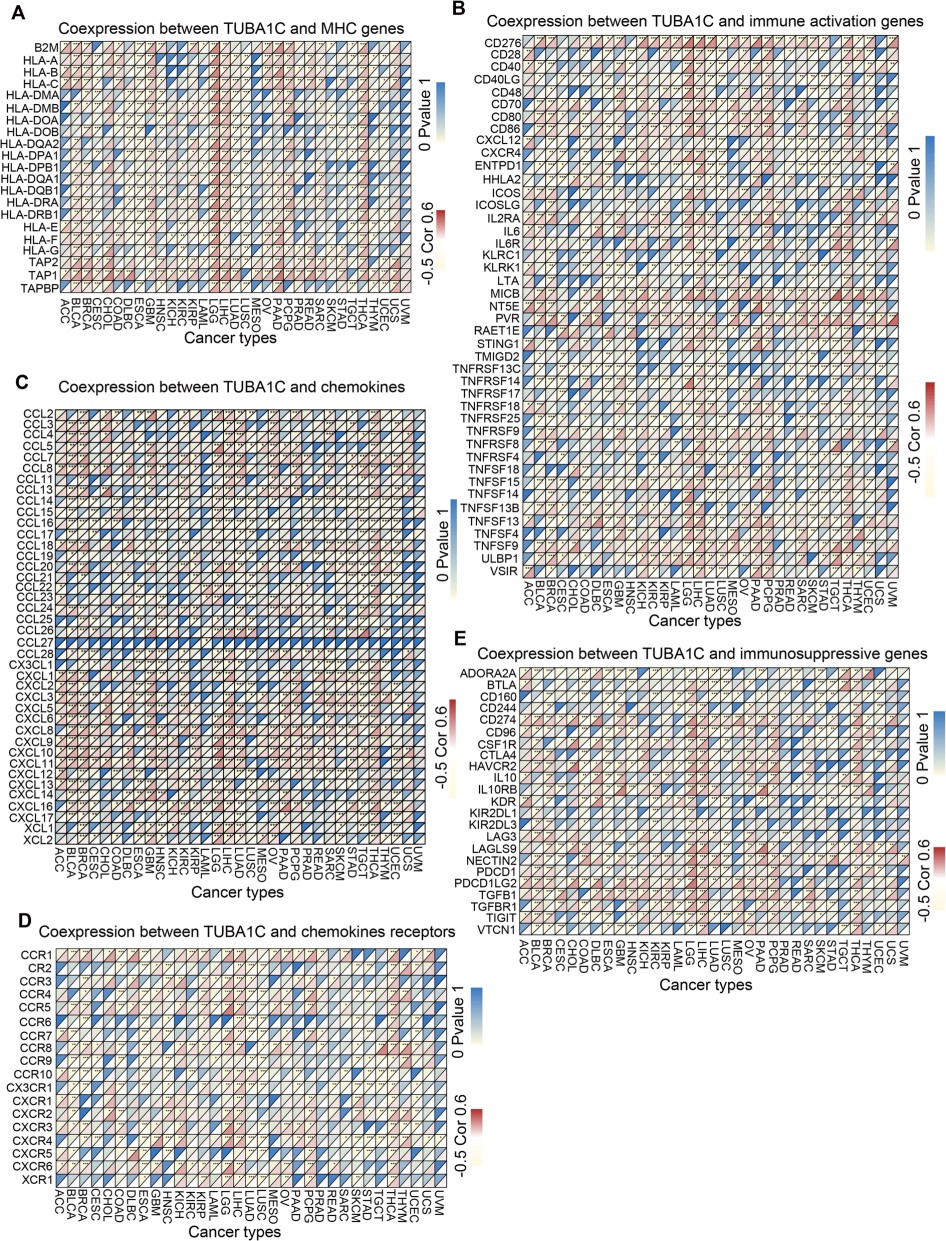
Association of TUBA1C expression and immune-associated genes in various cancers. **A** TUBA1C was coexpressed with MHC genes. **B** TUBA1C was coexpressed with immune activation genes. **C** TUBA1C was coexpressed with chemokines. **D** TUBA1C was coexpressed with chemokine receptors. **E** TUBA1C was coexpressed with immunosuppressive genes

Further gene coexpression analysis was carried out between TUBA1C and immune-associated genes, including those encoding MHC molecules, chemokines, chemokine receptors, and proteins related to immune activation and immunosuppression, in 33 cancers. The heatmaps revealed that most immune-associated genes except CCL27 were coexpressed with TUBA1C and that the main immune-associated genes were positively related to TUBA1C in LGG, LIHC, PCPG and THCA (Fig. 12). TUBA1C was coexpressed with MHC genes in several cancer types, particularly BLCA, ESCA, LGG, LICH and THCA (Fig. 12A). We also found that TUBA1C was coexpressed with immune activation genes in all cancer types and with immunosuppressive genes in most cancers except uveal melanoma (UVM) (Fig. 12B, E). TUBA1C was coexpressed with most chemokines except CCL27, and coexpression of TUBA1C with chemokines and chemokine receptors was observed in most cancers except UVM (C, D).

**Fig. 13:**
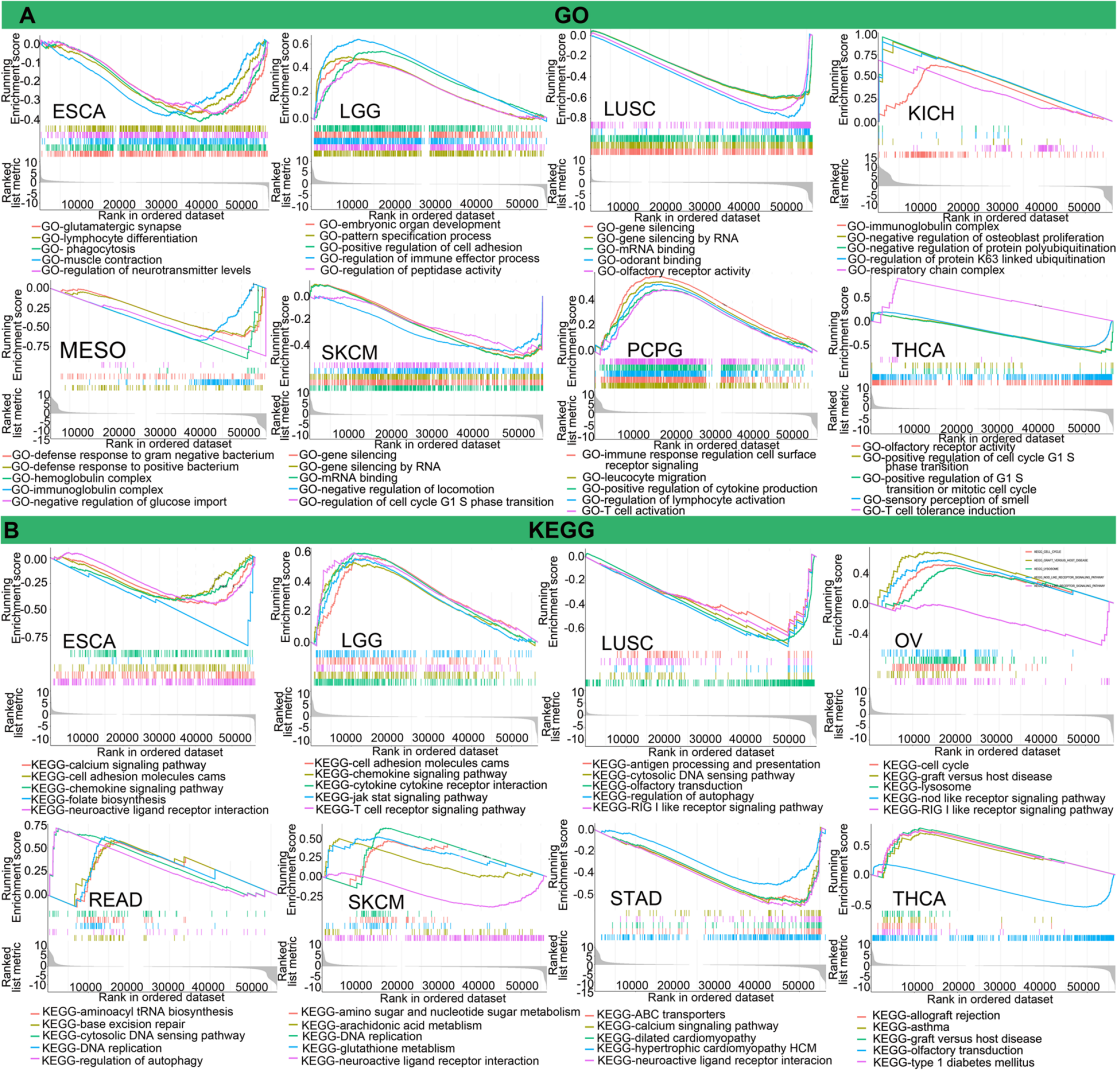
Relationship between TUBA1C expression and immune-associated pathways in diverse cancers. **A** TUBA1C was associated with several GO functional annotations in ESCA, LGG, LUSC, KICH, MESO, SKCM, PCPG and THCA. **B** TUBA1C was associated with several KEGG pathway annotations in ESCA, LGG, LUSC, OV, READ, SKCM, STAD and THCA

To explore the underlying mechanism of action of TUBA1C, we conducted GO term and KEGG pathway enrichment analyses of TUBA1C (Fig. 13). Our results suggested that TUBA1C participated in several immune-associated pathways in the GO database, such as regulation of lymphocyte activation, positive regulation of cytokine production, and T-cell activation, in PCPG. In addition, it influenced immunoglobulin in KICH and MESO (Fig. 13A). In ESCO, TUBA1C was associated with lymphocyte differentiation in GO analysis and with chemokines in KEGG analysis. In LGG, there was a tight link between TUBA1C and the GO term regulation of immune receptor processes and between TUBA1C and the KEGG pathways chemokine signaling pathway and T-cell receptor signaling pathway (Fig. 13A, B). KEGG pathway analysis also indicated that TUBA1C mediated RIG-I-like receptor signalling in OV and mediated the regulation of autophagy, antigen processing and presentation and RIG-I-like receptor signalling in LUSC (Fig. 13B). Furthermore, TUBA1C was implicated in many biological pathways, including miRNA binding and gene silencing by RNA, and in many other pathways, such as allograft rejection, drug metabolism cytochrome p40, retinol metabolism, and neuroactive ligand receptor interaction.

### Expression level and prognostic value of TUBA1C in gliomas

**Fig. 14:**
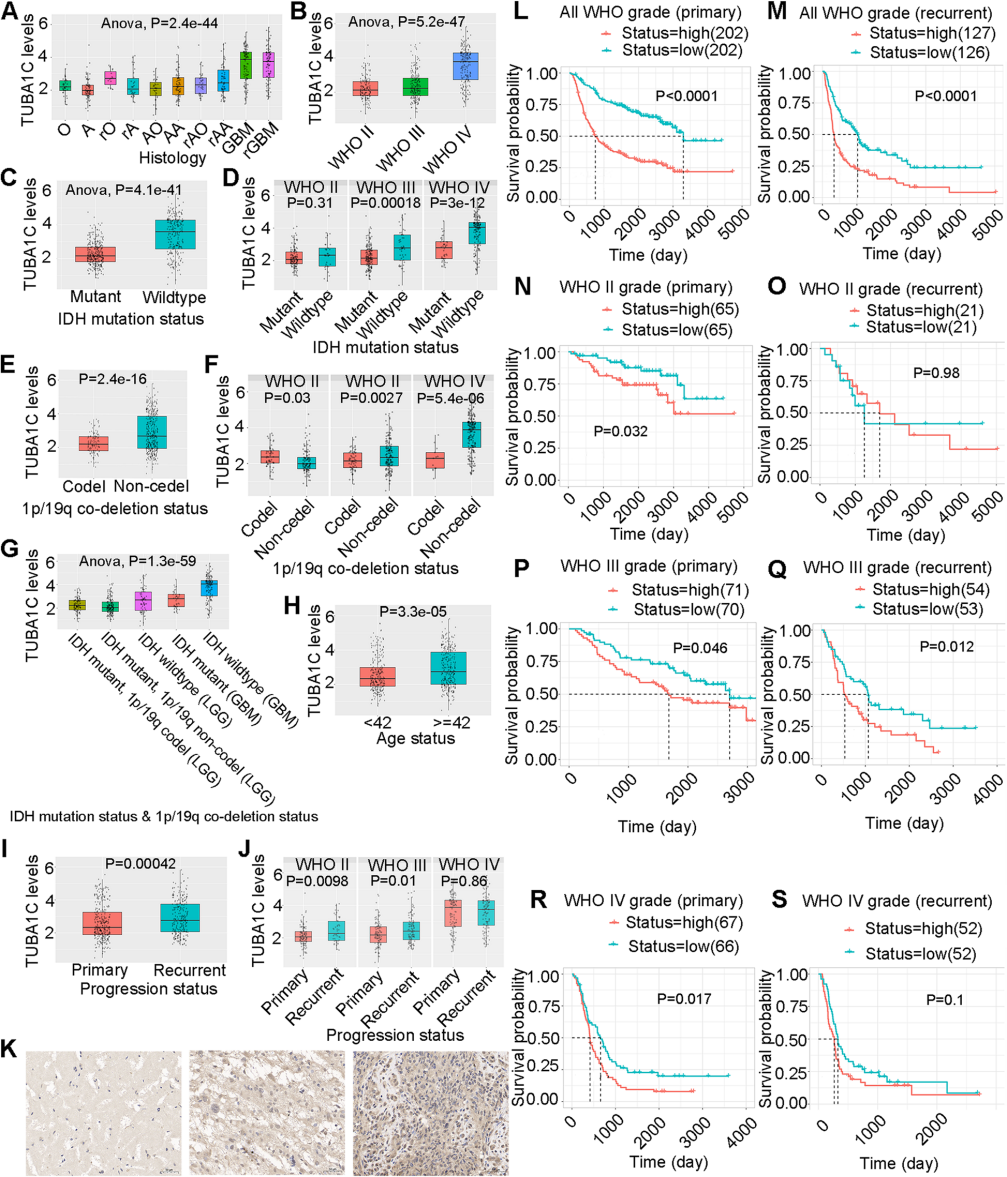
Expression levels of TUBA1C in glioma and normal tissues. TUBA1C expression in gliomas of different histology (**A**) and different WHO subtypes (**B**). TUBA1C expression in patients with different IDH mutation statuses (**C**) and different WHO subtypes (**D**). TUBA1C expression in patients with different 1p/19q codeletion statuses (**E**) and different WHO subtypes (**F**). TUBA1C expression in patients with different IDH mutation statuses and 1p/19q codeletion statuses (**G**) and different ages (**H**). TUBA1C expression in patients with different progression statuses (**I**) and different WHO subtypes (**J**). K TUBA1C expression in normal (left), low-grade glioma (middle) and high-grade glioma (right) tissue. Scale bars = 50 μm. Survival probability in the groups with different TUBA1C expression levels in all WHO subtypes of primary glioma (**L**) and recurrent glioma (**M**). Survival probability in the groups with different TUBA1C expression levels in WHO grade II primary glioma (**N**) and recurrent glioma (**O**). Survival probability in the groups with different TUBA1C expression levels in WHO grade III primary glioma (**P**) and recurrent glioma (**Q**). Survival probability in the groups with different TUBA1C expression levels in WHO grade IV primary glioma (**R**) and recurrent glioma (**S**)

We further investigated the expression of TUBA1C in gliomas and its impact on the prognosis of patients with gliomas. Figure 14A shows TUBA1C expression in gliomas of different histologies in the CGGA dataset. TUBA1C expression was highest in primary glioblastoma (pGBM) and recurrent pGBM (rGBM) and lowest in astrocytoma (A). In anaplastic oligodendrocytoma (AO) and anaplastic astrocytoma (AA), TUBA1C was expressed at moderate levels. Overall, TUBA1C expression increased in gliomas as WHO classification increased. In addition, TUBA1C expression was elevated in relapsed A, O, AO, and AA, similar to the results shown in Fig. 14I and Fig. 14J. Figure 14B further confirmed this phenomenon. We further found that TUBA1C expression was lower in the IDH mutant state than in the wild type (Fig. 14C), and similar results were found in WHO II, III and IV (Fig. 14D). In addition, TUBA1C expression was lower in patients with IDH mutation than in those with wild-type IDH (Fig. 14E), and similar results were found for WHO grades III and IV (Fig. 14F). In IDH wildtype LGG, the TUBA1C expression was higher than in LGG with IDH mutation and 1p/19q codeletion (Fig. 14G). Moreover, TUBA1C expression was higher in patients older than 42 years than in those aged < 42 years (Fig. 14H). Subsequently, we collected normal, LGG and GBM tissues for immunohistochemical analysis. The results showed that the IHC staining intensity was strongest in GBM (Fig. 14K, right), moderate in LGG (Fig. 14K, middle) and weakest in normal tissues (Fig. 14K, left). In primary gliomas, the probability of survival was higher in the TUBA1C low expression group than in the TUBA1C high expression group in all WHO grades (Fig. 14L, *p* < 0.0001) and in the WHO grade II (Fig. 14N, *p* = 0.032), WHO grade III (Fig. 14P, *p* = 0.032) and WHO grade IV (Fig. 14R, *p* = 0.017) subclassifications. In recurrent glioma, the probability of survival was higher in the TUBA1C low expression group in all WHO grades (Fig. 14M, *p* < 0.0001) and the WHO grade III (Fig. 14Q, *p* = 0.012) subclassification. In WHO II and IV recurrent glioma, the expression of TUBA1C was not related to the probability of survival (Fig. 14O, *p* = 0.98; Fig. 14S, *p* = 0.1).

## Discussion

Pancancer analysis can shed light on the expression, function and mechanism of genes in different cancers, providing new ideas and targets for improving the therapeutic efficacy in and prognosis of cancers. Microtubules, a main component of eukaryotic cells, play an important role in dynamic aggregation and depolymerization through cell division and replication [28], and TUBA1C is a subtype of α-tubulin. TUBA1C was reported to be related to cell proliferation and the cell cycle in diverse cancers [29] and was associated with the progression of several cancers [30], whereas no pancancer analysis has been performed for TUBA1C. Our research revealed that TUBA1C was significantly upregulated in 18 cancers, and immunohistochemistry confirmed consistent results for the protein expression levels of TUBA1C in cancers. Previous studies have shown that TUBA1C can predict poor outcome in PDAC [6] by modulating cell cycle signalling pathways to induce apoptosis, in LUAD [5] and LGG [11] by affecting TIICs in the tumour microenvironment, and in HCC [7] by cell cycle signalling pathways. Moreover, it was found that the combination of GTSE1 and TUBA1C predicted a 100% probability of triple-negative breast cancer (TNBC) in whites; NRF1, TUBA1B and BAX with EFNA4, and NRF1 and BTRC predicted a 100% probability of TNBC in blacks, confirming the potential of TUBA1C as a diagnostic marker [31]. In our research, an association of TUBA1C expression and poor prognosis (OS, DSS, DFI and PFI) of patients was observed, particularly in LGG, LIHC, MESO and KIRC. Notably, TUBA1C was highly expressed in KIRC and is related to OS in KIRC patients, suggesting that TUBA1C has promise as a diagnostic marker for KIRC. Moreover, we found that TUBA1C was correlated with the clinicopathological stage of cancers, which helps to determine the degree of malignancy of tumours. In BRCA, HNSC, KICH, KIRC, LUAD, MESO and READ, TUBA1C was expressed at higher levels in stage IV than in stage I tumours.

In recent years, ICIs have been a hot topic in the treatment of patients with advanced cancer [32], but predictive biomarkers are needed. The results of studies in certain cancers indicate that TMB can predict the clinical response to ICI therapy [33]. It has also been confirmed that patients with MSI-high colorectal cancers benefit significantly from ICI treatment compared to patients with MSI-low status [34]. Therefore, we investigated the association between TUBA1C expression and TMB and MSI. It was shown that TUBA1C affected TMB in 16 cancers and affected MSI in 8 cancers, suggesting that the efficacy of ICI therapy can be predicted by TUBA1C expression. We can assume that in cancers where TUBA1C expression is positively correlated with TMB or MSI, a high TUBA1C level predicts a good outcome and prognosis for immunotherapy.

Tumours are strongly influenced by the surrounding normal tissue, forming a special ecological niche called the TME [35]. The TME consists of stromal cells, fibroblasts, endothelial extracellular, innate and adaptive immune cells. Cytokines within the TME manipulate immune function, causing malfunction of the immune response and ultimately leading to tumour progression [36]. In addition, the TME is widely accepted to be a major player in the response to immunotherapy [37]. Therefore, it is essential to explore the relationship between TUBA1C and the TME. A higher immune score or stromal score denotes more immune or matrix components in the TME [38]. Our results showed that TUBA1C expression was significantly negatively related to the immune components of the TME in ESCA and positively related to the immune components of GBM, LGG, PCPG and THCA. In addition, TUBA1C expression was positively related to the stromal components of the TME in GBM and LGG and negatively related to the stromal components in ESCA, STAD and TGCT.

Moreover, we investigated the association between TUBA1C expression and immune cell infiltration in the TMB. Memory T cells are involved in specific and acquired immunity and play a fundamental role in the immune system [39]. TUBA1C expression was negatively related to memory CD4 T-cell infiltration in COAD, STAD and TGCT but positively associated in BRCA in our study. Regulatory T cells are specialized to suppress excessive immune activation and maintain immune homeostasis [40]. TUBA1C expression was negatively correlated with regulatory T cell infiltration in ESCA. Tumours manipulate neutrophils early in their differentiation process to change the state of the tumour by creating a different phenotype and functional polarization state [41]. TUBA1C expression was positively related to neutrophil infiltration in BLCA, HNSC, KIRC and SKCM. Macrophage infiltration in solid tumours is linked to poor prognosis and is correlated with chemoresistance in most cancers [42]. TUBA1C expression was positively related to M1 macrophage infiltration in CESC, KIRP, LGG and UCEC, with M2 macrophage infiltration in LUSC and with M0 macrophage infiltration in SARC. Mast cells interact with the immune and nonimmune components of the TMB to regulate tissue homeostasis and immune responses and act as antitumor agents [43]. TUBA1C expression was negatively correlated with mast cell infiltration in LUAD and THYM. Dendritic cells are natural mediators of antigen delivery, control immune tolerance and immune responses and are an important target for generating immunity against cancer [44]. We observed a positive correlation between TUBA1C and dendritic cell infiltration in THCA.

In addition, TUBA1C was coexpressed with immune-related genes encoding chemokine proteins, immune activators, immunosuppressive factors, chemokines and MHC. Moreover, we performed mechanistic analysis of TUBA1C using the GO and KEGG pathway analyses and found that TUBA1C was associated with several immune-associated pathways, such as regulation of lymphocyte activation, positive regulation of cytokine production, T cell activation, immunoglobulin, lymphocyte differentiation, immune receptor process, RIG-I-like receptor signaling and chemokine signaling. This suggests that TUBA1C may influence cancer progression and prognosis by affecting the tumour immune response.

Moreover, we validated the expression and role of TUBA1C in gliomas. Our study showed that TUBA1C was highly expressed in glioma types with high malignancy and in recurrent gliomas. In addition, TUBA1C expression was highest in WHO IV gliomas, intermediate in WHO III gliomas and lowest in WHO II gliomas. In addition, TUBA1C expression was reduced in patients with both IDH mutation and 1p/19q codeletion. Moreover, TUBA1C overexpression was observed in older patients (age ≥ 42) and predicted a poor outcome. Immunohistochemical analysis also confirmed that TUBA1C expression was higher in high-grade gliomas than in low-grade gliomas.

## Conclusion

In summary, the current study shows that upregulated TUBA1C is related to poor prognosis in cancer patients, is associated with a higher tumour grade, affects tumour sensitivity to and the efficacy of ICI therapy, influences immune cell infiltration in the TME, and interacts with immune-associated genes and pathways in cancers. Therefore, TUBA1C is a potential prognostic marker and may play an important role in immunotherapy.

## Supplementary Information

**Additional file 1: Figure S1.** The correlation between TUBA1C and immune cell infiltration in CIBERSOR. **Figure S2.** The correlation between TUBA1C and immune cell infiltration in EPIC. **Figure S3.** The correlation between TUBA1C and immune cell infiltration in MCPCOUNTER. **Figure S4.** The correlation between TUBA1C and immune cell infiltration in QUANTISEQ. **Figure S5.** The correlation between TUBA1C and immune cell infiltration in TIMER. **Figure S6.** The correlation between TUBA1C and immune cell infiltration in XCELL.

## Data Availability

The datasets supporting the conclusions of this article are available on the UCSC Xena (https://xena.ucsc.edu/), Oncomine (https://www.oncomine.org/), TCGA (https://tcga.xenahubs.net), the HPA database (https://www.proteinatlas.org/) and the CGGA database (http://www.cgga.org.cn/) websites. The datasets generated and/or analyzed during the current study are also available from the corresponding authors upon reasonable request in compliance with ethical standards.
