# Supplementary material for: The oncogenic role of tubulin alpha-1c chain in human tumours
Source: BMC Cancer. 2022 May 6;22:498. doi: 10.1186/s12885-022-09595-0 (PMC9074327; doi:10.1186/s12885-022-09595-0)
Supplement: Supplementary file 1 — Additional file 1: Figure S1. The correlation between TUBA1C and immune cell infiltration in CIBERSOR. Figure S2. The correlation between TUBA1C and immune cell infiltration in EPIC. Figure S3. The correlation between TUBA1C and immune cell infiltration in MCPCOUNTER. Figure S4. The correlation between TUBA1C and immune cell infiltration in QUANTISEQ. Figure S5. The correlation between TUBA1C and immune cell infiltration in TIMER. Figure S6. The correlation between TUBA1C and immune cell infiltration in XCELL. [file 12885_2022_9595_MOESM1_ESM.zip › Additional file 1.docx]

**Additional file 1: Figure S1** The correlation between TUBA1C and immune cell infiltration in CIBERSOR. **Figure S2** The correlation between TUBA1C and immune cell infiltration in EPIC. **Figure S3** The correlation between TUBA1C and immune cell infiltration in MCPCOUNTER. **Figure S4** The correlation between TUBA1C and immune cell infiltration in QUANTISEQ. **Figure S5** The correlation between TUBA1C and immune cell infiltration in TIMER. **Figure S6** The correlation between TUBA1C and immune cell infiltration in XCELL.
